# Supplementary material for: Generation of a Novel High-Affinity Antibody Binding to PCSK9 Catalytic Domain with Slow Dissociation Rate by CDR-Grafting, Alanine Scanning and Saturated Site-Directed Mutagenesis for Favorably Treating Hypercholesterolemia
Source: Biomedicines. 2021 Nov 27;9(12):1783. doi: 10.3390/biomedicines9121783 (PMC8698692; doi:10.3390/biomedicines9121783)
Supplement: Supplementary file 1 [file biomedicines-09-01783-s001.zip › biomedicines-1448633-supplementary.pdf]

Supplementary Figures

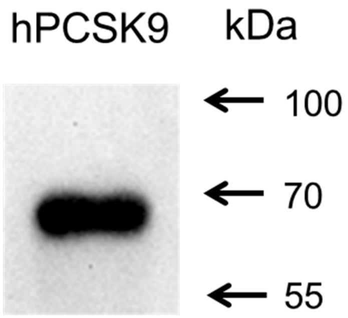

Figure S1. Purified hPCSK9 was identified by Western blot using the rabbit anti-human PCSK9 antibody (Cat# ab181142, 1:3000).

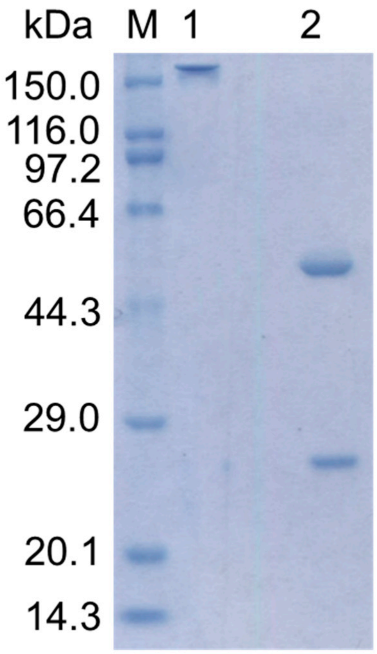

Figure S2. 10% (w/v) SDS-PAGE analysis of the purified m5E12. The purified antibody m5E12 was analyzed under nonreducing condition (lane 1) and reducing condition (lane 2). M, molecular weight marker.

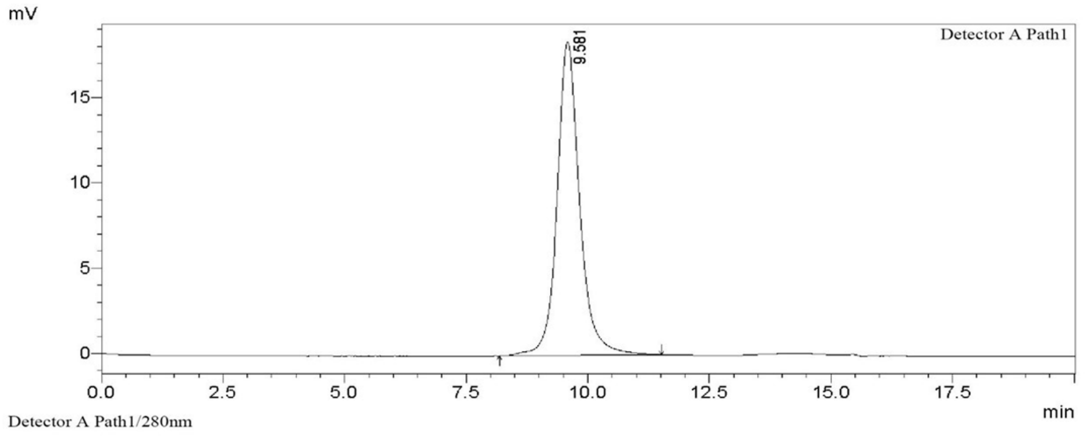

Figure S3. Purity analysis of m5E12. The purity of purified m5E12 was analyzed by Size-exclusion high-performance liquid chromatography (SE-HPLC) using a Shodex PROTEIN KW-802.5 column (SHOWA DENKO K.K., Japan) on a Shimadzu LC-2010 HPLC system according to the manufacturer's instructions.

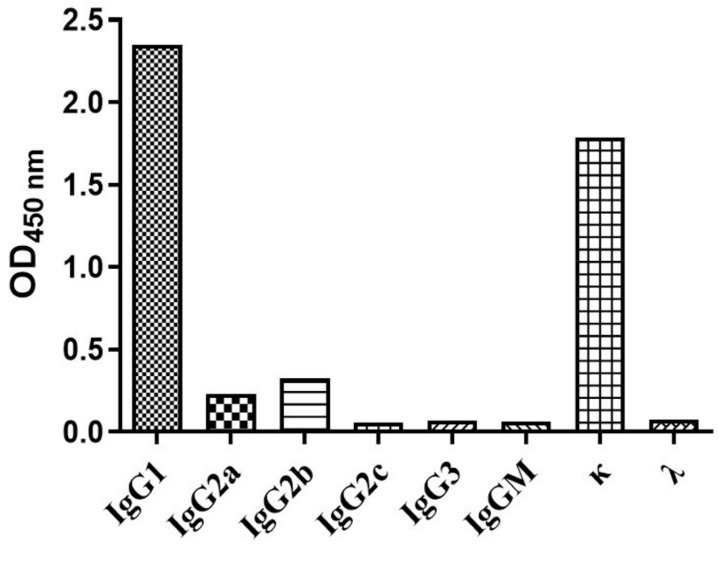

Figure S4. IgG isotype identification of m5E12. The isotype of m5E12 was analyzed using the mouse mAb isotyping kit according to the manufacture's manual, and the result showed that m5E12 was an IgG1/kappa mouse monoclonal antibody.

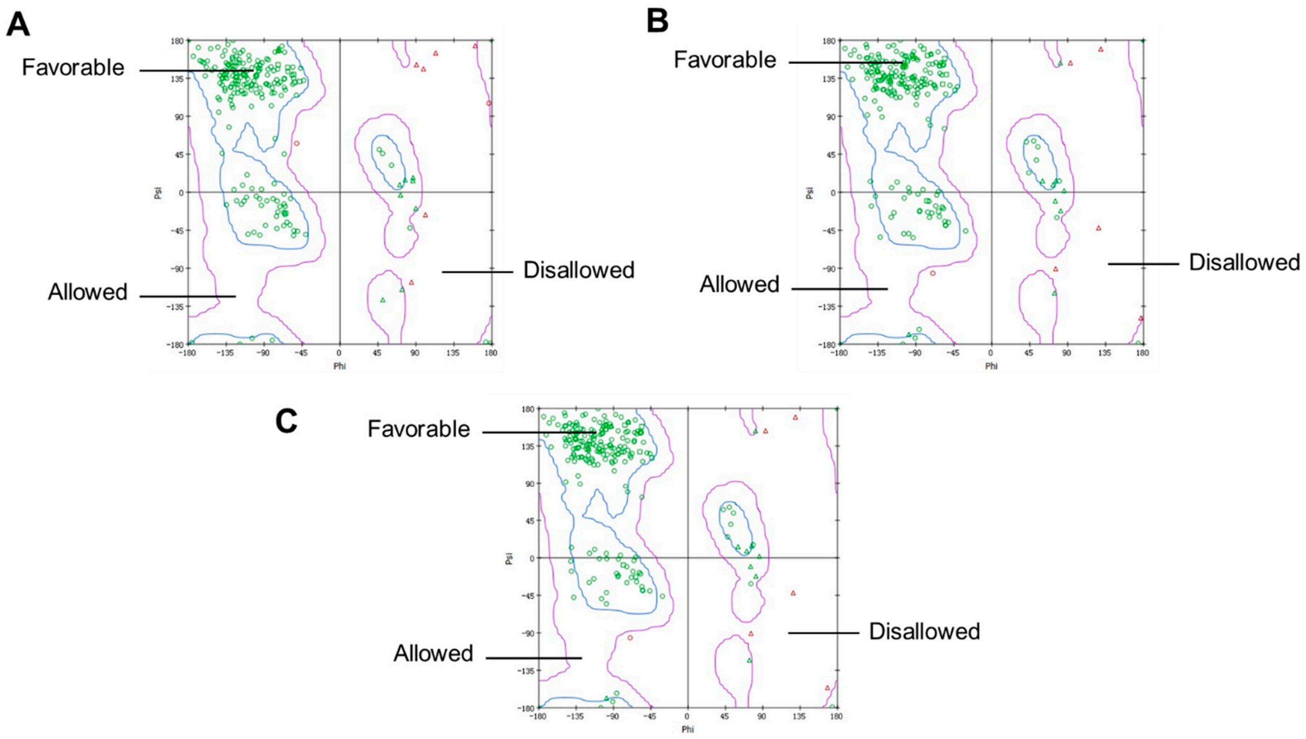

Figure S5. Ramachandran plots for modelled scFvs. **(A-B)** Ramachandran plot assessment of the modelled stereochemical structures of m5E12scfv **(A)**, h5E12scfv **(B)** and h5E12scFv-bm **(C)**. Triangles represent Glycine, squares represent proline, circles represent all other residues. 96.7%, 97.1%, 97.1% residues of m5E12scFv, h5E12scFv and h5E12scFv-bm, respectively, were distributed in the favored and allowed regions.

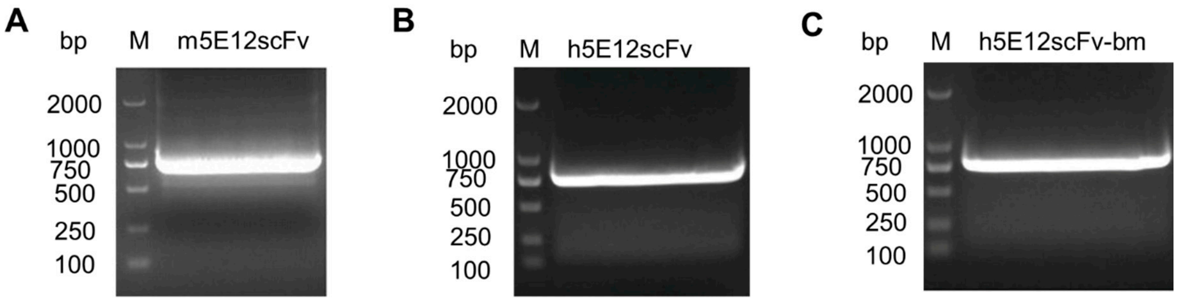

Figure S6. PCR-amplified DNA fragments of scFvs. (A) PCR-amplified DNA fragments of m5E12scFv. (B) PCR-amplified DNA fragments of h5E12scFv. **(C)** PCR-amplified DNA fragments of h5E12scFv-bm **(C)**. The size of scFvs were about 720 bp.

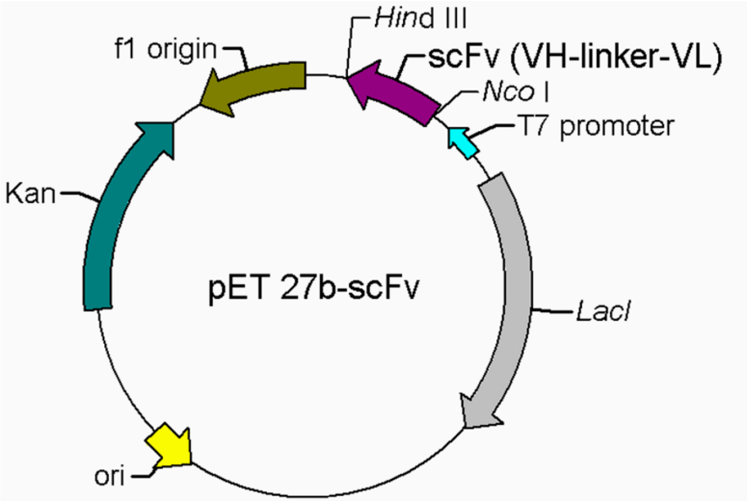

Figure S7. Schematic representation of plasmids expressing anti-hPCSK9 scFv. The restriction sites (*Nco* I/*Hin*d III) used for the construction of the vector are indicated.

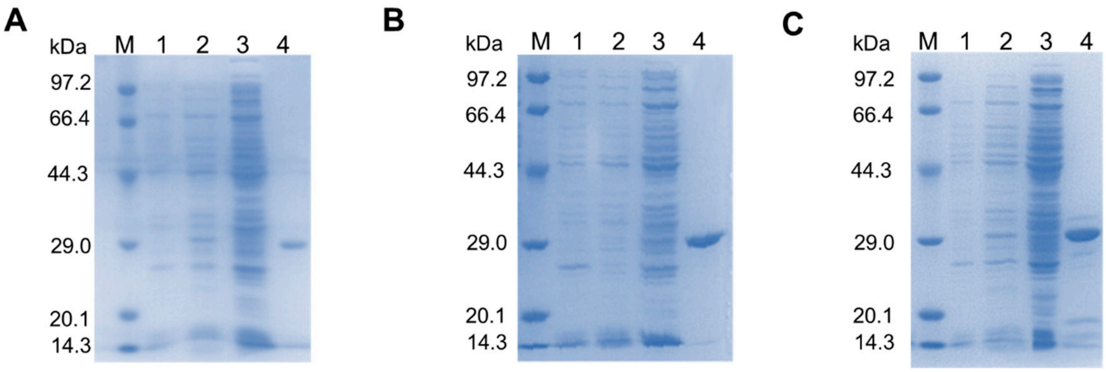

Figure S8. SDS-PAGE analysis of purified scFvs. **(A)** 12% (w/v) SDS-PAGE analysis of the purified m5E12scfv. **(B)** 12% (w/v) SDS-PAGE analysis of the purified h5E12scfv. **(C)** 12% (w/v) SDS-PAGE analysis of the purified h5E12scFv-bm **(C)**. Lane M: molecular weight marker; Lane 1, induced cell lysates of *E. coli* BL21(DE3) containing pET-27b; Lane 2, non-induced cell lysates; Lane 3, Periplasmic extracts containing scFvs; Lane 4, purified scFvs with an expected molecular weight of 29 kDa.

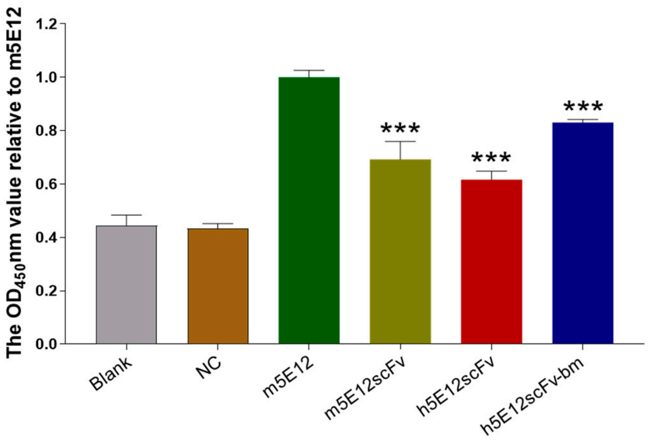

Figure S9. Competitive ELISA for determining binding specificity of scFvs in comparison with parental antibody m5E12. hPCSK9-coated microplates were incubated with 0.5 μg/ml m5E12 mixed with or without 300 μg/ml scFvs (m5E12scFv, h5E12scFv or h5E12scFv-bm) at 37°C for 2 h. An equal volume of the vehicle (3% BSA in PBS) was used as the negative control, and wells without any treatment were taken as blank control. The result suggested that the scFvs could compete against parental antibody m5E12 to bind to hPCSK9, and the binding ability of m5E12scFv and h5E12scFv were stronger than h5E12scFv-bm. NC, negative control. ***P<0.001 vs. m5E12 group.

```
                                                  HCDR1                      HCDR2                                                        HCDR3
                  1        10        20        30         40             50        60                70        80        90         100       110          120
h5E12-scFv        QVQLVQSGAEVKKPGASVKVSCKAS GYTFTSYWMH WVRQAPGQGLEWMG EINPSNGRTDHSEKFKN RVTMTRDTSTSTVYMELSSLRSEDTAVYYCAR FHYDYDYFD YWGQGTLVTVSS GGG
h5E12scFv-L230A   ------------------------- ---------- -------------- ----------------- -------------------------------- --------- ------------ ---
h5E12scFv-L230S   ------------------------- ---------- -------------- ----------------- -------------------------------- --------- ------------ ---
h5E12scFv-L230G   ------------------------- ---------- -------------- ----------------- -------------------------------- --------- ------------ ---
h5E12scFv-L230D   ------------------------- ---------- -------------- ----------------- -------------------------------- --------- ------------ ---
h5E12scFv-L230V   ------------------------- ---------- -------------- ----------------- -------------------------------- --------- ------------ ---
h5E12scFv-L230I   ------------------------- ---------- -------------- ----------------- -------------------------------- --------- ------------ ---
h5E12scFv-L230C   ------------------------- ---------- -------------- ----------------- -------------------------------- --------- ------------ ---
h5E12scFv-L230P   ------------------------- ---------- -------------- ----------------- -------------------------------- --------- ------------ ---
h5E12scFv-L230Q   ------------------------- ---------- -------------- ----------------- -------------------------------- --------- ------------ ---
h5E12scFv-L230M   ------------------------- ---------- -------------- ----------------- -------------------------------- --------- ------------ ---
h5E12scFv-L230N   ------------------------- ---------- -------------- ----------------- -------------------------------- --------- ------------ ---
h5E12scFv-L230W   ------------------------- ---------- -------------- ----------------- -------------------------------- --------- ------------ ---
h5E12scFv-L230Y   ------------------------- ---------- -------------- ----------------- -------------------------------- --------- ------------ ---
h5E12scFv-L230R   ------------------------- ---------- -------------- ----------------- -------------------------------- --------- ------------ ---
h5E12scFv-L230T   ------------------------- ---------- -------------- ----------------- -------------------------------- --------- ------------ ---
h5E12scFv-L230F   ------------------------- ---------- -------------- ----------------- -------------------------------- --------- ------------ ---
h5E12scFv-L230H   ------------------------- ---------- -------------- ----------------- -------------------------------- --------- ------------ ---
h5E12scFv-L230E   ------------------------- ---------- -------------- ----------------- -------------------------------- --------- ------------ ---
h5E12scFv-L230K   ------------------------- ---------- -------------- ----------------- -------------------------------- --------- ------------ ---
```

```
                  Linker                             LCDR1                         LCDR2                                          LCDR3
                          130        140       150        160       170       180        190       200       210       220        230       240
h5E12-scFv        GSGGGGSGGGGS DIVMTQSPSSLSASVGDRVTITC KASRNVGNSVG WYQQKPGKAPKLLIY SASYRYS GVPSRFSGSGSGTDFTLTISSLQPEDFATYYC QQYNRYPLTF GQGTKVEIK
h5E12scFv-L230A   ------------ ----------------------- ----------- --------------- ------- -------------------------------- -------A-- ---------
h5E12scFv-L230S   ------------ ----------------------- ----------- --------------- ------- -------------------------------- -------S-- ---------
h5E12scFv-L230G   ------------ ----------------------- ----------- --------------- ------- -------------------------------- -------G-- ---------
h5E12scFv-L230D   ------------ ----------------------- ----------- --------------- ------- -------------------------------- -------D-- ---------
h5E12scFv-L230V   ------------ ----------------------- ----------- --------------- ------- -------------------------------- -------V-- ---------
h5E12scFv-L230I   ------------ ----------------------- ----------- --------------- ------- -------------------------------- -------I-- ---------
h5E12scFv-L230C   ------------ ----------------------- ----------- --------------- ------- -------------------------------- -------C-- ---------
h5E12scFv-L230P   ------------ ----------------------- ----------- --------------- ------- -------------------------------- -------P-- ---------
h5E12scFv-L230Q   ------------ ----------------------- ----------- --------------- ------- -------------------------------- -------Q-- ---------
h5E12scFv-L230M   ------------ ----------------------- ----------- --------------- ------- -------------------------------- -------M-- ---------
h5E12scFv-L230N   ------------ ----------------------- ----------- --------------- ------- -------------------------------- -------N-- ---------
h5E12scFv-L230W   ------------ ----------------------- ----------- --------------- ------- -------------------------------- -------W-- ---------
h5E12scFv-L230Y   ------------ ----------------------- ----------- --------------- ------- -------------------------------- -------Y-- ---------
h5E12scFv-L230R   ------------ ----------------------- ----------- --------------- ------- -------------------------------- -------R-- ---------
h5E12scFv-L230T   ------------ ----------------------- ----------- --------------- ------- -------------------------------- -------T-- ---------
h5E12scFv-L230F   ------------ ----------------------- ----------- --------------- ------- -------------------------------- -------F-- ---------
h5E12scFv-L230H   ------------ ----------------------- ----------- --------------- ------- -------------------------------- -------H-- ---------
h5E12scFv-L230E   ------------ ----------------------- ----------- --------------- ------- -------------------------------- -------E-- ---------
h5E12scFv-L230K   ------------ ----------------------- ----------- --------------- ------- -------------------------------- -------K-- ---------
```

Figure S10. Amino acid alignment of h5E12scFv variants. The amino acid sequence of h5E12scFv was aligned with the sequence of its corresponding saturated mutagenesis variants. Dashes (-) represent the same residue as h5E12scFv.

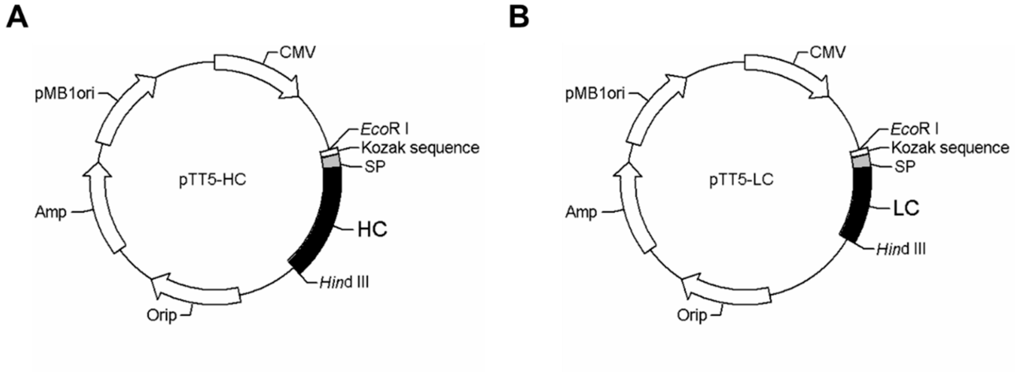

Figure S11. Plasmid maps. **(A)** Schematic representation of plasmids expressing full-length heavy chains. **(B)** Schematic representation of plasmids expressing full-length light. The restriction sites (*Eco*R I/*Hin*d III) used for the construction of the vector are indicated. Kozak, Kozak consensus sequence; SP, signal peptide; HC, heavy chain; LC, light chain.

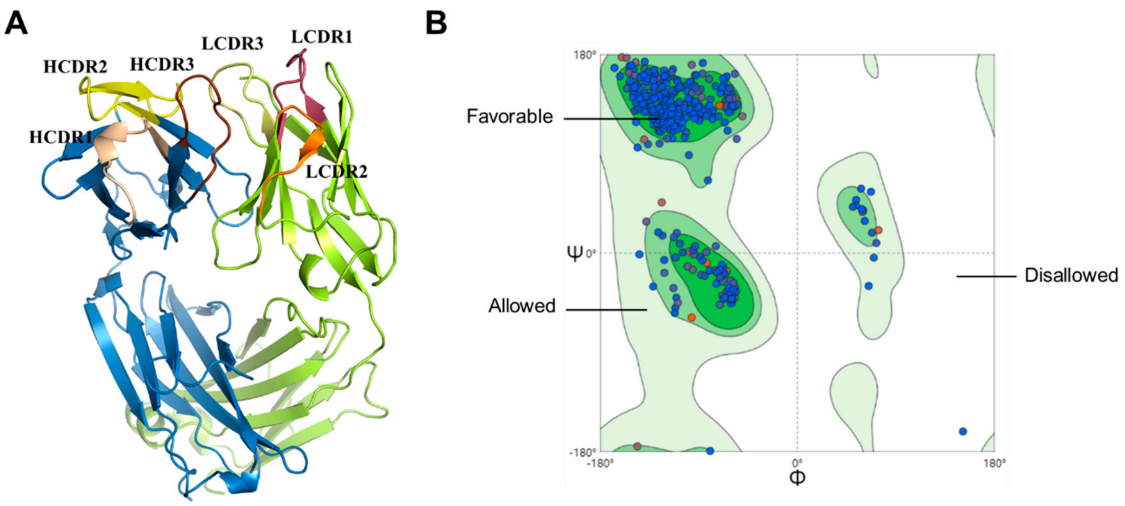

Figure S12. Homology modeling and validation of the antibody Fab fragment of h5E12-L230G. **(A)** Three-dimensional homology model of the antibody Fab fragment of h5E12-L230G. The heavy chains were colored in skyblue, and the light chains were in limon. The CDR loops were colored with HCDR1 in wheat, HCDR2 in yellow, HCDR3 in chocolate, LCDR1 in raspberry, LCDR2 in orange, and LCDR3 in paleyellow. **(B)** Ramachandran plot assessment of the antibody Fab fragment of h5E12-L230G. 97.91% of the residues were in the most favorable and allowed regions.

Supplementary Tables

Table S1. PCR primers for amplification of V_H and V_L genes

Primer	Sequence (5′-3′)
V_H-F[1]	CCGGAATTCSARGTNMAGCTGSAGSAGTC
V_H-R[1]	CCCAAGCTTATAGACAGATGGGGGTGTCGTTTTGGC
V_L-F[2]	CCGGAATTCGAYATTGTGMTSACMCARWCTMCA
V_L-R[2]	CCCAAGCTTGGATACAGTTGGTGCAGCATC

[1] V_H-F and V_H-R were primers for V_H, [2] V_L-F and V_L-R were primers for V_L

Table S2. PCR primers for amplification of humanized scFvs

Primer	Sequence (5′-3′)
m-F[1]	CATGCCATGGATGAAGTTCAGCTGGAGCAGTCAG
m-R[1]	CCCAAGCTTTCA*ATGGTGATGGTGATGGTG*TTTCAGCTCCAGCTTGGTCC
h-F[2]	CATGCCATGGATCAGGTGCAGCTGGTGCAGTCTG
bm-F[3]	CATGCCATGGATGAAGTTCAGCTGGTGCAGTCAG
h-R/bm-R[2 3]	CCCAAGCTTTCA*ATGGTGATGGTGATGGTG*TTTGATCTCCACCTTGGTCC

[1] m-F and m-R were primers for m5E12scFv, [2] h-F and h-R were primers for h5E12scFv, [3] bm-F and bm-R were primers for h5E12scFv-bm. The protective bases and restriction sites are marked with underline, the His-tag bases are marked in italics.

Table S3. PCR primers for scanning mutagenesis of h5E12scFv

Primer	Sequence (5′-3′)
F99A-F	ACGGCCGTGTATTACTGTGCGAGA**GC**CCACTATGATTACGACTACTTTGATTAC
F99A-R	**GC**TCTCGCACAGTAATACACGGCCGTGTCCTCAGATCTCAGGCTGCTG
H100A-F	CTGTGCGAGATTC**GC**CTATGATTACGACTACTTTGATTACTGG
H100A-R	**GC**GAATCTCGCACAGTAATACACGGCCGTGTCC
D102A-F	TATTACTGTGCGAGATTCCACTATGCTTACGACTACTTTGATTACTGGGGCCAGG
D102A-R	**G**CATAGTGGAATCTCGCACAGTAATACACGGCCGTGTCCTCAGATCTCAGGCTG
D104A-F	GATTCCACTATGATTACG**C**CTACTTTGATTACTGGGGCCAGGGGACCCTGGTC
D104A-R	AAAGTAG**G**CGTAATCATAGTGGAATCTCGCACAGTAATACACGGCCGTGTCCTCAG
F106A-F	AGATTCCACTATGATTACGACTAC**GC**TGATTACTGGGGCCAGGGGACCCTGGTC
F106A-R	**GC**GTAGTCGTAATCATAGTGGAATCTCGCACAGTAATACACGGCCGTGTCCTC
D107A-F	TTACGACTACTTTG**C**TTACTGGGGCCAGG
D107A-R	**G**CAAAGTAGTCGTAATCATAGTGGAATCTCGC
R227A-F	ACTTACTATTGTCAGCAATATAAC**GC**GTATCCGCTCACGTTTGGCCAGGGGACC
R227A-R	**GC**GTTATATTGCTGACAATAGTAAGTTGCAAAATCTTCAGGCTGCAGGCTGC
P229A-F	ATTGTCAGCAATATAACAGGTAT**G**CGCTCACGTTTGGCCAGGGGACCAAGGTGG
P229A-R	CG**C**ATACCTGTTATATTGCTGACAATAGTAAGTTGCAAAATCTTCAGGCTGC
L230A-F	TGTCAGCAATATAACAGGTATCCG**GC**CACGTTTGGCCAGGGGACCAAGGTGGAGATC
L230A-R	TG**GC**CGGATACCTGTTATATTGCTGACAATAGTAAGTTGCAAAATCTTCAGGCTGC
T231A-F	AATATAACAGGTATCCGCTC**G**CGTTTGGCCAGGGGACCAAGGTGGAGATCAAAC
T231A-R	AAACG**C**GAGCGGATACCTGTTATATTGCTGACAATAGTAAGTTGCAAAATCTTC

The bold letters represent the mutated bases.

Table S4. PCR primers for saturated mutagenesis variants of h5E12scFv

Primer	Sequence (5′-3′)
L230S-F	ACGGCCGTGTATTACTGTGCGAG**AGC**CCACTATGATTACGACTACTTTGATTAC
L230S-R	T**GCT**CGGATACCTGTTATATTGCTGACAATAGTAAGTTGCAAAATCTTCAGGCTGC
L230G-F	TGTCAGCAATATAACAGGTATCCG**GGC**ACGTTTGGCCAGGGGACCAAGGTGGAGATC
L230G-R	T**GCC**CGGATACCTGTTATATTGCTGACAATAGTAAGTTGCAAAATCTTCAGGCTGC
L230D-F	TGTCAGCAATATAACAGGTATCCG**GAC**ACGTTTGGCCAGGGGACCAAGGTGGAGATC
L230D-R	T**GTC**CGGATACCTGTTATATTGCTGACAATAGTAAGTTGCAAAATCTTCAGGCTGC
L230V-F	TGTCAGCAATATAACAGGTATCCG**GTT**ACGTTTGGCCAGGGGACCAAGGTGGAGATC
L230V-R	T**AAC**CGGATACCTGTTATATTGCTGACAATAGTAAGTTGCAAAATCTTCAGGCTGC
L230I-F	TGTCAGCAATATAACAGGTATCCG**ATT**ACGTTTGGCCAGGGGACCAAGGTGGAGATC
L230I-R	T**AAT**CGGATACCTGTTATATTGCTGACAATAGTAAGTTGCAAAATCTTCAGGCTGC
L230C-F	TGTCAGCAATATAACAGGTATCCG**TGC**ACGTTTGGCCAGGGGACCAAGGTGGAGATC
L230C-R	T**GCA**CGGATACCTGTTATATTGCTGACAATAGTAAGTTGCAAAATCTTCAGGCTGC
L230P-F	TGTCAGCAATATAACAGGTATCCG**CCG**ACGTTTGGCCAGGGGACCAAGGTGGAGATC
L230P-R	T**CGG**CGGATACCTGTTATATTGCTGACAATAGTAAGTTGCAAAATCTTCAGGCTGC
L230O-F	TGTCAGCAATATAACAGGTATCCG**CAG**ACGTTTGGCCAGGGGACCAAGGTGGAGATC
L230O-R	T**CTG**CGGATACCTGTTATATTGCTGACAATAGTAAGTTGCAAAATCTTCAGGCTGC
L230M-F	TGTCAGCAATATAACAGGTATCCG**ATG**ACGTTTGGCCAGGGGACCAAGGTGGAGATC
L230M-R	T**CAT**CGGATACCTGTTATATTGCTGACAATAGTAAGTTGCAAAATCTTCAGGCTGC
L230N-F	TGTCAGCAATATAACAGGTATCCG**AAC**ACGTTTGGCCAGGGGACCAAGGTGGAGATC
L230N-R	T**GTT**CGGATACCTGTTATATTGCTGACAATAGTAAGTTGCAAAATCTTCAGGCTGC
L230W-F	TGTCAGCAATATAACAGGTATCCG**TGG**ACGTTTGGCCAGGGGACCAAGGTGGAGATC
L230W-R	T**CCA**CGGATACCTGTTATATTGCTGACAATAGTAAGTTGCAAAATCTTCAGGCTGC
L230Y-F	TGTCAGCAATATAACAGGTATCCG**TAC**ACGTTTGGCCAGGGGACCAAGGTGGAGATC
L230Y-R	T**GTA**CGGATACCTGTTATATTGCTGACAATAGTAAGTTGCAAAATCTTCAGGCTGC
L230R-R	T**ACG**CGGATACCTGTTATATTGCTGACAATAGTAAGTTGCAAAATCTTCAGGCTGC
L230T-F	TGTCAGCAATATAACAGGTATCCG**ACC**ACGTTTGGCCAGGGGACCAAGGTGGAGATC
L230T-R	T**GGT**CGGATACCTGTTATATTGCTGACAATAGTAAGTTGCAAAATCTTCAGGCTGC
L230F-F	TGTCAGCAATATAACAGGTATCCG**TTT**ACGTTTGGCCAGGGGACCAAGGTGGAGATC
L230F-R	T**AAA**CGGATACCTGTTATATTGCTGACAATAGTAAGTTGCAAAATCTTCAGGCTGC
L230H-F	TGTCAGCAATATAACAGGTATCCG**CAT**ACGTTTGGCCAGGGGACCAAGGTGGAGATC
L230H-R	T**ATG**CGGATACCTGTTATATTGCTGACAATAGTAAGTTGCAAAATCTTCAGGCTGC
L230E-F	TGTCAGCAATATAACAGGTATCCG**GAA**ACGTTTGGCCAGGGGACCAAGGTGGAGATC
L230E-R	T**TTC**CGGATACCTGTTATATTGCTGACAATAGTAAGTTGCAAAATCTTCAGGCTGC
L230K-F	TGTCAGCAATATAACAGGTATCCG**AAA**ACGTTTGGCCAGGGGACCAAGGTGGAGATC
L230K-R	T**TTT**CGGATACCTGTTATATTGCTGACAATAGTAAGTTGCAAAATCTTCAGGCTGC

The bold letters represent the mutated bases.

Table S5. PCR primers for amplification of full-length antibodies

Primer	Sequence (5′-3′)
H-F0	***CCGGAATTC***GCCGCCACCATGGATTGGACCTGGAGATTTCTGTTTGTGGTGGCCGC
H-F1	TTCTGTTTGTGGTGGCCGCCGCCACAGGCGTGCAGTCTCAGGTGCAGCTGGTGCAG
H-R1	CACGGATGGGCCCTTTGTGCTGGCCGAGGAGACGGTGACCAGGGTCC
H-F2	GGACCCTGGTCACCGTCTCCTCGGCCAGCACAAAGGGCCCATCCGTG
H-R2	***CCCAAGCTT***TGGATACAGTTGGTGCAGCATCAGCCCGTTTC
L-F0	***CCGGAATTC***GCCGCCACCATGGACATGAGGGTGCCAGCTCAGCTGCTGGGACTGC
L-F1	CAGCTGCTGGGACTGCTGCTGCTGTGGCTGTCCGGAGCTAGGTGCGATATTGTGAT
L-R1	CTTGGAGCGGCCACGGTTCTTTTGATCTCCACCTTGGTCC
L-F2	GGACCAAGGTGGAGATCAAAAGAACCGTGGCCGCTCCAAG
L-R2	***CCCAAGCTT***TGGATACAGTTGGTGCAGCATCAGCC

The protective bases and restriction sites are marked in italics, the bases encoding the kozak sequence are marked with underline. The 5' end of H-F0 and L-F0 contained *Eco*R I restriction site (GAATTC), the 5' end of H-R2 and L-R2 contained *Hin*d III restriction site (AAGCTT).

Table S6. Kinetic parameters of murine and humanized scFvs binding to hPCSK9

scFvs	k_{on} (M⁻¹s⁻¹)	k_{off} (s⁻¹)	K_D (M) [1]
m5E12scFv	1.02×10^4	6.37×10^{-3}	6.22×10^{-7}
h5E12scFv	6.32×10^3	1.08×10^{-3}	1.71×10^{-7}
h5E12scFv-bm	1.50×10^4	1.00×10^{-2}	6.69×10^{-7}
Ali-scFv	8.93×10^3	4.55×10^{-4}	5.08×10^{-8}

scFv, single-chain variable fragment. [1] The equilibrium dissociation constant (K_D) was calculated using the formula $K_D = k_{off}/k_{on}$.

Table S7. Kinetic parameters of h5E12-L230G and Alirocumab binding to hPCSK9

mAbs	k_{on} ($M^{-1}s^{-1}$)	k_{off} (s^{-1})	K_D (M) [1]
h5E12-L230G	2.81×10^4	4.84×10^{-5}	1.72×10^{-9}
Alirocumab	8.04×10^4	6.87×10^{-5}	8.54×10^{-10}

mAb, Monoclonal antibody. [1] The equilibrium dissociation constant (K_D) was calculated using the formula $K_D = k_{off}/k_{on}$.

Table S8. Protein-protein contacts between h5E12-L230G and PCSK9

Number	Type	Chain	Position	Residue	Chain	Position	Residue	CDR
1	HB	PCSK9	168	Ala168	HC	55	Asn55	HCDR2
2	HB	PCSK9	168	Ala168	HC	33	Trp33	HCDR1
3	HB	PCSK9	179	Leu179	LC	50	Ser50	LCDR2
4	HB	PCSK9	181	Glu181	LC	49	Tyr49	LCDR2
5	HB	PCSK9	197	Glu197	HC	108	Tyr108	HCDR3
6	HB	PCSK9	198	Gly198	LC	56	Ser56	LCDR2
7	ION	PCSK9	199	Arg199	HC	107	Asp107	HCDR3
8	ION	PCSK9	199	Arg199	HC	107	Asp107	HCDR3
9	HB	PCSK9	200	Val200	LC	56	Ser56	LCDR2
10	HB	PCSK9	202	Val202	LC	54	Arg54	LCDR2
11	HB	PCSK9	237	Arg237	HC	25	Ser25	FR1
12	HB	PCSK9	238	Asp238	HC	28	Thr28	HCDR1
13	HB	PCSK9	238	Asp238	HC	28	Thr28	HCDR1
14	HB	PCSK9	243	Lys243	HC	100	His100	HCDR3
15	HB	PCSK9	246	Ser246	HC	103	Tyr103	HCDR3
16	HB	PCSK9	279	Pro279	LC	53	Tyr53	LCDR2
17	HB	PCSK9	401	Ser401	HC	102	Asp102	HCDR3

HB: Hydrogen bond; ION: Ionic bond; HC: heavy chain; LC: light chain; FR: Framework region.
